# Cost–utility analysis of liraglutide compared with sulphonylurea or sitagliptin, all as add-on to metformin monotherapy in Type 2 diabetes mellitus

**DOI:** 10.1111/j.1464-5491.2011.03429.x

**Published:** 2012-03

**Authors:** M J Davies, B D Chubb, I C Smith, W J Valentine

**Affiliations:** 1 Department of Cardiovascular Sciences, University of LeicesterLeicester; 2 Novo Nordisk LtdCrawley, West Sussex, UK; 3 Ossian Health Economics and CommunicationsBasel, Switzerland

**Keywords:** cost-effectiveness, liraglutide, Type 2 diabetes, UK

## Abstract

**Aim:**

To investigate the cost-effectiveness of liraglutide as add-on to metformin vs. glimepiride or sitagliptin in patients with Type 2 diabetes uncontrolled with first-line metformin.

**Methods:**

Data were sourced from a clinical trial comparing liraglutide vs. glimepiride, both in combination with metformin, and a clinical trial comparing liraglutide vs. sitagliptin, both as add-on to metformin. Only the subgroup of patients in whom liraglutide was added to metformin monotherapy was included in the cost–utility analysis. The CORE Diabetes Model was used to simulate outcomes and costs with liraglutide 1.2 and 1.8 mg vs. glimepiride and vs. sitagliptin over patients’ lifetimes. Treatment effects were taken directly from the trials. Costs and outcomes were discounted at 3.5% per annum and costs were accounted from a third-party payer (UK National Health System) perspective.

**Results:**

Treatment with liraglutide 1.2 and 1.8 mg resulted, respectively, in mean increases in quality-adjusted life expectancy of 0.32 ± 0.15 and 0.28 ± 0.14 quality-adjusted life years vs. glimepiride, and 0.19 ± 0.15 and 0.31 ± 0.15 quality-adjusted life years vs. sitagliptin, and was associated with higher costs of £3003 ± £678 and £4688 ± £639 vs. glimepiride, and £1842 ± £751 and £3224 ± £683 vs. sitagliptin, over a patient’s lifetime. Both liraglutide doses were cost-effective, with incremental cost-effectiveness ratios of £9449 and £16 501 per quality-adjusted life year gained vs. glimepiride, and £9851 and £10 465 per quality-adjusted life year gained vs. sitagliptin, respectively.

**Conclusions:**

Liraglutide, added to metformin monotherapy, is a cost-effective option for the treatment of Type 2 diabetes in a UK setting.

## Introduction

Diabetes is among the most common chronic illnesses worldwide, with Type 2 diabetes mellitus accounting for approximately 90% of all cases [1]. Type 2 diabetes is progressive and is characterized by increased insulin resistance, generally associated with obesity, and deteriorating β-cell function, resulting in chronic hyperglycaemia. As the disease progresses, so do the micro- and macrovascular complications associated with it, which have a negative impact on the quality of life of patients and pose a huge economic burden to the health system [2,3]. For example, in the UK, the cost of Type 2 diabetes accounts for 7–12% of the total National Health Service (NHS) expenditure [4].

The risk of micro- and macrovascular complications is strongly associated with hyperglycaemia, and each reduction of 11 mmol/mol (1%) in HbA_1c_ significantly reduces the risk of developing these complications in patients with Type 2 diabetes [5]. In the UK, the National Institute for Health and Clinical Excellence (NICE) recently issued recommendations for the optimum management of Type 2 diabetes, taking into consideration the effectiveness, safety and cost-effectiveness of the available treatments (NICE, 2009) [6]. NICE recommends lifestyle modifications and metformin as first-line therapy, with the subsequent stepwise additions of a sulphonylurea and insulin. A thiazolidinedione or a dipeptidyl peptidase-4 (DPP-4) inhibitor may be considered as second-line options in place of a sulphonylurea if there is a significant risk of hypoglycaemia, or if a sulphonylurea is contraindicated or not tolerated. Sitagliptin (a DPP-4 inhibitor) or a thiazolidinedione can be considered as third-line therapy in place of insulin if insulin is unacceptable. Exenatide may also be considered as a third-line option in combination with metformin and a sulphonylurea in patients with a BMI above 35 kg/m^2^ and problems associated with high weight, or BMI under 35 kg/m^2^ if insulin is unacceptable because of occupational implications, or if weight loss would benefit other co-morbidities [6]. The place of liraglutide (Victoza®; Novo Nordisk A/S, Bagsvaerd, Denmark) in therapy has also been evaluated recently by NICE [7].

Recommendations advocate the use of liraglutide 1.2 mg daily in triple therapy (with metformin and a sulphonylurea or metformin and a thiazolidinedione) under the same conditions described for exenatide, and in dual therapy (with metformin or a sulphonylurea) if metformin or sulphonylureas and thiazolidinediones or DPP-4 inhibitors cannot be tolerated or are contraindicated [7]. The American Diabetes Association and the European Association for the Study of Diabetes issued similar recommendations in a consensus algorithm based on effectiveness and safety data from clinical trials and on clinical experience, taking into account benefits, risks and costs of the different available treatments [8]. In clinical trials, glucagon-like peptide 1 (GLP-1) receptor agonists, such as liraglutide and exenatide, have been shown to reduce HbA_1c_ to at least the same, and often to a greater, extent than traditional oral hypoglycaemic agents and both Glucagon-like peptide (GLP-1) receptor agonists and DPP-4 inhibitors such as Sitagliptin, have the additional advantages of reducing the risk of hypoglycaemia, as their insulinotropic effect is glucose-dependent, and inducing weight loss (in the case of GLP-1 receptor agonists) or being weight-neutral (in the case of DPP-4 inhibitors) [9]. Additionally, GLP-1 receptor agonists have been shown to have a positive effect on systolic blood pressure [9]. Despite these advantages, sulphonylureas continue to be the preferred second-line choice after metformin, with incretin-based therapies only recommended as second- or third-line therapies in special circumstances [6,8]. The fact that incretin-based therapies are considered more expensive may contribute to these therapies not being recommended more widely.

Liraglutide is a GLP-1 analogue approved in 2009 for use in Europe, including the UK. Because of its recent approval, studies evaluating the cost-effectiveness of liraglutide are scarce. The aim of our study was to investigate the cost-effectiveness of liraglutide as add-on to metformin compared with glimepiride or sitagliptin in patients failing treatment with first-line metformin.

## Patients and methods

### Data sources

The cost–utility evaluation carried out in this study is based on patients who participated in two studies performed as part of the phase III clinical development programme for liraglutide: a study comparing liraglutide vs. glimepiride (LEAD-2 study), both in combination with metformin, and a study comparing liraglutide vs. sitagliptin, both also in combination with metformin [10,11]. In the LEAD-2 study, adults with Type 2 diabetes and HbA_1c_ between 53 and 97 mmol/mol (7–11%) (if previously treated with oral hypoglycaemic agent monotherapy for at least 3 months) or HbA_1c_ between 53 and 86 mmol/mol (7–10%) (if previously treated with oral hypoglycaemic agent combination therapy for at least 3 months) were included. Additional inclusion criteria were age between 18 and 80 years and BMI ≤ 40 kg/m^2^. To facilitate recruitment into the trial, previous treatment with other oral anti-diabetes drugs, as monotherapy or in combination, was allowed [10]. However, only the subgroup of patients in which liraglutide or glimepiride was added to metformin monotherapy (approximately 30% of the total trial population) was included in the cost–utility analysis presented here, as this was considered to be more reflective of actual clinical practice. In the liraglutide vs. sitagliptin study, adults with Type 2 diabetes, previously treated with metformin monotherapy for at least 3 months and with HbA_1c_ between 58 and 86 mmol/mol (7.5–10.0%) were included. Additional inclusion criteria were age between 18 and 80 years and BMI ≤ 45 kg/m^2^ [11]. Demographic characteristics of the patients enrolled in these studies have previously been described [10,11].

### The CORE Diabetes Model

The cost–utility evaluation presented here was carried out using the CORE Diabetes Model, details of which have been published previously by Palmer *et al*. [12]. The CORE diabetes model is a validated [13] non-product-specific policy analysis tool based on a series of 15 sub-models that simulate major complications of diabetes: cardiovascular disease, stroke, neuropathy, foot ulcer/amputation, eye disease, nephropathy, hypoglycaemia, lactic acidosis and non-specific mortality [12]. For each sub-model, a combination of semi-Markov model structure and Monte Carlo simulations were used. This structure allows patients to develop multiple complications within each model cycle and over the simulation period. The model projects outcomes for populations, considering baseline cohort characteristics, past history of complications, concomitant medications, current and future diabetes management, screening strategies and changes in physiological variables over time. In this way, incidence of complications, life expectancy, quality-adjusted life expectancy and total costs within populations can be calculated. The results can be expressed in terms of quality-adjusted life years (QALYs) gained and incremental cost-effectiveness ratios, i.e. the cost per QALY gained. An incremental cost-effectiveness ratio threshold of £20 000–30 000 per QALY gained is generally considered to represent good value for money in the UK [14].

### Simulation cohorts and treatments

A simulated cohort of patients was defined (Table 1), with baseline demographics and complications taken from the respective clinical trial used in the analysis. Treatment effects with liraglutide (1.2 and 1.8 mg) vs. glimepiride and liraglutide (1.2 and 1.8 mg) vs. sitagliptin were taken directly from the clinical trials (Table 2). Treatment duration was set to 5 years, after which basal insulin therapy was started in an attempt to replicate clinical practice. Simulations were run over patients’ lifetimes to capture all events and complications related to the progression of Type 2 diabetes.

**Table 1:** Baseline patient characteristics in the liraglutide vs. glimepiride and liraglutide vs. sitagliptin studies

	Liraglutide vs. glimepiride (*n* = 263)*	Liraglutide vs. sitagliptin (*n* = 635)
Patient demographics		
Age (years)	55.8 (9.0)	55.3 (9.2)
Diabetes duration (years)	6 (5)	6 (5)
Proportion male (%)	54.2	52.9
Risk factors		
HbA_1c_ (mmol/mol)	67 (8.9)	68 (6.5)
(%)	8.3 (1.1)	8.4 (0.8)
Systolic blood pressure (mmHg)	130.6 (14.0)	132.2 (14.5)
Body mass index (kg/m^2^)	31.0 (4.7)	32.8 (5.2)
Total cholesterol (mmol/l)	4.88 (1.12)	4.09 (1.14)
HDL-C (mmol/l)	1.29 (0.33)	1.16 (0.31)
LDL-C (mmol/l)	3.11 (0.89)	2.65 (0.82)
Triglycerides (mmol/l)	2.19 (1.66)	2.38 (2.22)
Current smoker (%)†	19.3*	19.3
Cigarettes/day^**†**^	10	10
Alcohol consumption (Oz/week)†	5	5
Ethnic group (%)		
Caucasian	88.5	90.0
Black	2.4	7.5
Hispanic	0	0
Native American	0	0.5
Asian/Pacific Islander	9.1	0.2

* Subgroup of patients from LEAD-2 in which liraglutide or glimepiride was added to metformin monotherapy.

†Smoking status from Scottish Diabetes Survey 2007 [24]; cigarette and alcohol consumption are estimates.The numbers in parentheses indicate standard deviation.

**2:** Treatment effects in the liraglutide vs. glimepiride (previous metformin monotherapy subgroup only) and liraglutide vs. sitagliptin studies

	Liraglutide vs. glimepiride			Liraglutide vs. sitagliptin		
		
Risk factor	Liraglutide 1.2 mg + metformin *n* = 91	Liraglutide 1.8 mg + metformin *n* = 83	Sulphonylurea + metformin *n* = 89	Liraglutide 1.2 mg + metformin *n* = 214	Liraglutide 1.8 mg + metformin *n* = 211	Sitagliptin 100 mg + metformin *n* = 210
Change in HbA_1c_ (mmol/mol) (%)	−13.7 (11.2)	−14.2 (10.8)	−12.7 (10.6)	−13.1 (11.0)	−16.4 (9.7)	−10.0 (11.6)
	−1.25 (1.02)	−1.30 (0.99)	−1.16 (0.97)	−1.24 (1.04)	−1.50 (0.89)	−0.90 (1.04)
Change in systolic blood pressure (mmHg)	−3.33 (12.90)	−1.18 (12.70)	2.26 (12.65)	−0.55 (13.23)	−0.72 (13.14)	−0.94 (13.17)
Change in total cholesterol (mmol/l)*	−0.02 (0.82)	−0.30 (0.80)	0.09 (0.08)	−0.03 (0.82)	−0.17 (0.80)	−0.02 (0.80)
Change in LDL-C (mmol/l)*	0.15 (0.68)	0.13 (0.67)	0.22 (0.67)	0.08 (0.69)	0.05 (0.67)	0.13 (0.68)
Change in HDL-C (mmol/l)*	0.02 (0.21)	−0.03 (0.20)	−0.02 (0.20)	0.00 (0.17)	0.00 (0.17)	0.00 (0.17)
Change in triglycerides (mmol/l)*	−0.44 (1.29)	−0.26 (1.26)	−0.25 (1.26)	−0.19 (1.42)	−0.43 (1.37)	−0.40 (1.38)
Change in BMI (kg/m^2^)	−0.64 (0.95)	−0.75 (1.11)	0.48 (3.69)	−1.00 (0.08)	−1.18 (0.08)	−0.34 (0.08)
Major hypo event rate (per 100 patient years)	0	0	0	1	0	0
Minor hypo event rate (per 100 patient years)	4.9	17.1	217.2	17.8	16.1	10.6

Data are mean (sd).

* The model accepts values in mg/dl. The following factors have been used to convert to mmol/l: 0.0259 for total cholesterol, LDL-C and HDL-C, and 0.0113 for triglycerides.

### Costs and utilities

Costs were accounted from a third-party payer (National Health Service) perspective. Where possible, unit costs for complications were derived from UK-specific published sources in patients with Type 2 diabetes and inflated to 2008 values, the latest available at the time of analysis, using the composite National Health Service price inflation index from the Personal Social Services Research Unit (PSSRU). A summary of the costs of medicines and complications is given in the Supporting Information (Table S1). The utilities used in the base case presented here are summarized in the Supporting Information (Table S2). The costs of medicines, self-monitored blood glucose testing equipment and needles were taken from the Monthly Index of Medical Specialities (MIMS) August 2009 [15]. Utilities and disutilities (i.e. measures of the impact on quality of life) associated with complications of diabetes were obtained from the literature and, where possible, taken from populations with Type 2 diabetes. Discount rates of 3.5% per annum for both costs and clinical outcomes were applied in the base case.

### Sensitivity analyses

To assess the impact of varying the key assumptions and outcomes used in the base-case analysis, several sensitivity analyses were performed: treatment duration was set to 3 and 8 years; an alternative weight progression was used in which, when treatment is switched, BMI reverts to baseline level and then increases as predicted with insulin treatment; discount rates were set to 0 and 6% for both costs and outcomes; and hypoglycaemia disutility was removed and also set to 0.0052, as used in the technology appraisal of insulin glargine carried out by NICE [18]. Additional analyses to investigate the contribution of individual clinical effects (weight, cholesterol and triglycerides, systolic blood pressure and HbA_1c_) to quality-adjusted life expectancy were also performed. The values used in the sensitivity analyses were derived from expert consensus or were previously used by, or recommended by, NICE in its *Guide to the Methods of Technology Appraisal* [16,17]. The results of these analyses are presented as approximate relative impacts of the base-case benefit. It should be noted that these values represent crude approximations (and therefore will not typically sum to 100%), as sensitivity analyses reflecting changes in multiple clinical variables have a complex impact on outcomes (in relation to the base case).

### Statistical methodology

A non-parametric bootstrapping approach was used for this health economic analysis. Using second-order Monte Carlo simulation, Type 2 diabetes progression was simulated in 1000 patients through the model 1000 times to calculate the mean and standard deviation of life expectancy, quality-adjusted life expectancy, and costs [12]. The results from the bootstrapped simulations were used to create cost-effectiveness acceptability curves.

## Results

### Base-case analyses

#### Liraglutide vs. glimepiride

**Figure 1:**
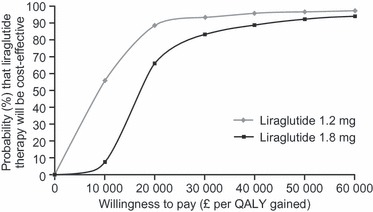
Cost-effectiveness acceptability curve of liraglutide vs. glimepiride, base case. QALY, quality-adjusted life year.

Treatment with liraglutide 1.2 and 1.8 mg resulted, respectively, in a mean increase in quality-adjusted life expectancy of 0.32 ± 0.15 QALYs and 0.28 ± 0.14 QALYs, and was associated with higher costs of £3003 ± £678 and £4688 ± £639 over a patient’s lifetime, compared with glimepiride. The estimated incremental cost-effectiveness ratios for liraglutide 1.2 and 1.8 mg vs. glimepiride were, respectively, £9449 and £16 501 per QALY gained (Table 3). At a willingness to pay of £20 000 per QALY gained, liraglutide 1.2 mg is a cost-effective treatment option in over 88% of cases, whereas liraglutide 1.8 mg is a cost-effective treatment option in over 65% of cases. If the willingness-to-pay threshold is increased to £30 000, the probability that the treatment will be cost-effective increases to over 93% for liraglutide 1.2 mg and 83% for liraglutide 1.8 mg (Fig. 1).

**3:** Results of the base-case analysis: quality-adjusted life years (QALYs), costs and incremental cost-effectiveness ratios (ICERs)

	Liraglutide vs. glimepiride				
	
	Liraglutide 1.2 mg+ metformin	Liraglutide 1.8 mg+ metformin	Sulphonylurea 4 mg+ metformin	Difference liraglutide 1.2 mg− sulphonylurea	Difference liraglutide 1.8 mg− sulphonylurea
QALYs	7.76 (0.11)	7.73 (0.10)	7.44 (0.11)	0.32 (0.15)	0.28 (0.14)
Costs (£)	22 122 (502)	23 807 (473)	19 119 (475)	3003 (678)	4688 (639)
ICER (£ per QALY)	—	—	—	9449	16 501

	Liraglutide vs. sitagliptin				
	
	Liraglutide 1.2 mg+ metformin	Liraglutide 1.8 mg+ metformin	Sitagliptin 100 mg+ metformin	Difference liraglutide 1.2 mg− sitagliptin	Difference liraglutide 1.8 mg− sitagliptin
QALYs	7.52 (0.11)	7.64 (0.11)	7.34 (0.11)	0.19 (0.15)	0.31 (0.15)
Costs (£)	21 793 (544)	23 175 (510)	19 951 (521)	1842 (751)	3224 (683)
ICER (£ per QALY)	—	—	—	9851	10 465

Data are mean (sd).

#### Liraglutide vs. sitagliptin

Compared with sitagliptin, mean increases in quality-adjusted life expectancy of 0.19 ± 0.15 QALYs and 0.31 ± 0.15 QALYs, and higher costs of £1842 ± £751 and £3224 ± £683 were associated with liraglutide 1.2 and 1.8 mg, respectively, over a patient’s lifetime. The estimated incremental cost-effectiveness ratios for liraglutide 1.2 and 1.8 mg vs. sitagliptin were, respectively, £9851 and £10 465 per QALY gained (Table 3). At a willingness to pay of £20 000, liraglutide 1.2 mg is a cost-effective treatment option in over 77% of cases, while liraglutide 1.8 mg is a cost-effective treatment option in over 85% of cases. The probability that the treatment will be cost-effective increases to 82% for liraglutide 1.2 mg and 92% for liraglutide 1.8 mg when the willingness-to-pay threshold is increased to £30 000.

### Sensitivity analyses

#### Liraglutide vs. glimepiride and liraglutide vs. sitagliptin

Decreasing the discount rate resulted in a lower incremental cost-effectiveness ratio with liraglutide 1.2 mg, while increasing the discount rate increased the incremental cost-effectiveness ratio. Reducing treatment duration from 5 to 3 years resulted in a lower incremental cost-effectiveness ratio for liraglutide 1.2 mg (Table 4). In the shorter treatment duration simulation, the full clinical benefit of liraglutide was achieved, but the cost was reduced as liraglutide pharmacy costs were only accounted for 3 years. Increasing treatment duration to 8 years resulted in a higher incremental cost-effectiveness ratio for liraglutide 1.2 mg, as, in this simulation, liraglutide pharmacy costs were accounted for 8 years, with the same clinical benefit of 5 years’ treatment. The length of liraglutide treatment for individual patients in a real-life setting will vary, but it is reassuring to note that treatment durations of 3, 5 and 8 years are all cost-effective at a willingness to pay of £20 000 per QALY gained (Table 4). Similar trends were observed for liraglutide 1.8 mg (data not shown).

**Table 4:** Results of the sensitivity analyses: quality-adjusted life years (QALYs), costs and incremental cost-effectiveness ratios (ICERs)

Liraglutide vs. glimepiride							

	QALYs (years)			Costs (£)			
			
Sensitivity analyses	Liraglutide 1.2 mg	Glimepiride 4 mg	Difference	Liraglutide 1.2 mg	Glimepiride 4 mg	Difference	ICER(£ per QALY gained)
Base case	7.76 (0.11)	7.44 (0.11)	0.32 (0.15)	22 122 (502)	19 119 (475)	3003 (678)	9449
3 years’ treatment	7.74 (0.11)	7.44 (0.11)	0.31 (0.15)	21 463 (501)	19 975 (477)	1488 (678)	4859
8 years’ treatment	7.78 (0.11)	7.45 (0.11)	0.33 (0.15)	22 983 (506)	18 005 (472)	4978 (679)	14 950
0% discount rate	10 924 (0.19)	10 418 (0.19)	0.51 (0.26)	34 374 (936)	30 985 (90.3)	3389 (1300)	6696
6% discount rate	6333 (0.10)	6090 (0.10)	0.243 (0.11)	17 108 (358)	14 289 (336)	2818 (476)	11 589
Alternative weight progression	7.71 (0.11)	7.48 (0.11)	0.23 (0.15)	22 122 (502)	19 119 (475)	3003 (678)	13 175
BMI disutility = −0.0061	8.04 (0.11)	7.77 (0.11)	0.27 (0.16)	22 122 (502)	19 119 (475)	3003 (678)	11 219
Hypoglycaemia disutility = −0.0052	7.74 (0.11)	7.41 (0.11)	0.33 (0.15)	22 122 (502)	19 119 (475)	3003 (678)	9010
Hypoglycaemia disutility = 0	7.80 (0.11)	7.51 (0.11)	0.29 (0.15)	22 122 (502)	19 119 (475)	3003 (678)	10 472

Liraglutide vs. sitagliptin							

	QALYs (years)			Costs (£)			
			
Sensitivity analyses	Liraglutide 1.2 mg	Sitagliptin 100 mg	Difference	Liraglutide 1.2 mg	Sitagliptin 100 mg	Difference	ICER (£ per QALY gained
Base case	7.52 (0.11)	7.34 (0.11)	0.19 (0.15)	21 793 (544)	19 951 (521)	1842 (751)	9851
3 years’ treatment	7.50 (0.10)	7.32 (0.11)	0.18 (0.14)	21 064 (532)	20 270 (521)	793 (737)	4321
8 years’ treatment	7.54 (0.11)	7.35 (0.11)	0.18 (0.15)	22 674 (534)	19 536 (520)	3138 (715)	16 497
0% discount rate	10.49 (0.18)	10.19 (0.19)	0.30 (0.25)	33 565 (971)	31 562 (938)	2003 (1,274)	6720
6% discount rate	6.16 (0.08)	6.02 (0.08)	0.14 (0.11)	16 922 (401)	15 170 (383)	1750 (528)	12 452
Alternative weight progression	7.44 (0.10)	7.30 (0.11)	0.13 (0.14)	21 793 (544)	19 951 (521)	1842 (715)	13 752
BMI disutility = −0.0061	7.86 (0.11)	7.70 (0.12)	0.16 (0.15)	21 793 (544)	19 951 (521)	1842 (715)	11 637
Hypoglycaemia disutility = −0.0052	7.50 (0.10)	7.31 (0.11)	0.19 (0.15)	21 793 (544)	19 951 (521)	1842 (715)	9852
Hypoglycaemia disutility = 0	7.55 (0.11)	7.36 (0.11)	0.19 (0.15)	21 793 (544)	19 951 (521)	1842 (715)	9686

Data are mean (sd).

#### Contribution of clinical effects to QALYs gained

The results of the additional analyses carried out to investigate the contribution of individual clinical effects (weight, cholesterol and triglycerides, systolic blood pressure and HbA_1c_) to QALYs showed that the gain in QALYs with liraglutide 1.2 mg over glimepiride is equally distributed between systolic blood pressure (32%), weight (30%) and cholesterol and triglycerides (27%), with only a smaller contribution from HbA_1c_ (11%). Conversely, the gain in QALYs with liraglutide 1.2 mg over sitagliptin arises mainly from improvements in HbA_1c_ (54%) and weight (44%). Cholesterol and triglycerides and systolic blood pressure changes had a negligible effect on QALYs gained (−3 and −1%, respectively). Additional Supporting Information may be found in the online version of this article.

## Discussion

The cost per QALY vs. glimepiride and vs. sitagliptin, for both doses of liraglutide investigated in this cost–utility modelling study (1.2 and 1.8 mg), ranged between £9000 and £16 000. Treatment with liraglutide costs more than with the comparators, but these increased costs were partially offset by reductions in the costs associated with complications, because the risk of developing complications decreases with liraglutide treatment as a result of its combined beneficial effects on body weight, blood glucose, systolic blood pressure and other cardiovascular risk factors. The values obtained lie below the threshold of £20 000–30 000 per QALY, indicating that liraglutide in combination with metformin monotherapy is a cost-effective option for the treatment of Type 2 diabetes compared with glimepiride or sitagliptin. The sensitivity analyses performed indicated that, in the liraglutide vs. glimepiride comparison, systolic blood pressure, weight and cholesterol were the key drivers of cost-effectiveness, with a relatively small contribution from HbA_1c_. This was to be expected, as both liraglutide and glimepiride treatment achieved similar HbA_1c_ reductions in the clinical trial on which this health economic evaluation is based, while liraglutide had a greater impact on reducing systolic blood pressure, weight and cholesterol compared with glimepiride [10]. In contrast, HbA_1c_ and weight were the key drivers of cost-effectiveness in the liraglutide vs. sitagliptin comparison, with only small effects from systolic blood pressure and cholesterol, reflecting the greater effect of liraglutide vs. sitagliptin on reducing HbA_1c_ and weight, and the comparable effects of both of these therapies on systolic blood pressure and cholesterol [11]. In the liraglutide vs. sitagliptin comparison, a preliminary subgroup analysis in which patients were stratified by baseline BMI (all > 30 or > 35 kg/m^2^) showed that the cost-effectiveness of liraglutide 1.2 mg vs. sitagliptin improved with increasing BMI, with incremental cost-effectiveness ratios of £9851, £7593 and £6125, respectively (see also Supporting Information, Table S3), probably because weight loss with liraglutide increases with increasing BMI [19]. This initial finding is interesting and may warrant further investigation at a later date. Treatment satisfaction was also assessed in the liraglutide vs. sitagliptin clinical trial using the Diabetes Treatment Satisfaction Questionnaire (DTSQ) and patients reported greater treatment satisfaction with liraglutide [11]. This result was not taken into consideration in the cost–utility analysis presented here. However, had it been, the cost-effectiveness of liraglutide vs. sitagliptin may have been even further enhanced, as treatment satisfaction could translate into greater adherence and improved clinical outcomes [20]. Furthermore, contrary to the perception that oral treatments are usually preferred to injections, there were no differences in the perceived convenience of treatment between sitagliptin and liraglutide [11].

**Figure 2:**
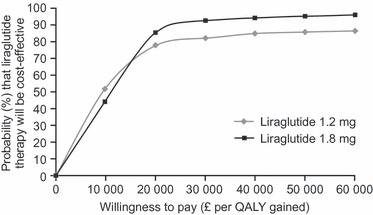
Cost-effectiveness acceptability curve of liraglutide vs. sitagliptin, base case. QALY, quality-adjusted life year.

To put the results of this economic evaluation into context, the cost per QALY of implementing liraglutide in combination with metformin therapy estimated in this study is in the same range as that estimated for implementing education programmes aimed at maximizing the benefits of diet and lifestyle interventions as reported in a recent study, which estimated a cost per QALY ranging from €10 000 to €39 000 [21]. However, a study that investigated the cost-effectiveness of the Diabetes Education and Self management for Ongoing and Newly Diagnosed (DESMOND) programme in UK patients newly diagnosed with Type 2 diabetes reported a lower cost per QALY of £2092 [22]. The estimated cost per QALY of adding pioglitazone to ongoing therapy in patients with Type 2 patients with a history of macrovascular disease and at high risk for further cardiovascular events was reported as £5396 vs. placebo after a mean treatment period of 3 years [23].The cost of adding sitagliptin to metformin monotherapy vs. the cost of adding a sulphonylurea appears to also be in the same range as the cost of adding liraglutide to metformin monotherapy reported here. An analysis to evaluate the cost of adding sitagliptin vs. sulphonylurea to metformin monotherapy in patients with Type 2 diabetes from six European countries (Austria, Finland, Portugal, Scotland, Spain and Sweden) and not reaching the International Diabetes Federation’s HbA_1c_ target of < 48 mmol/mol (< 6.5%) estimated costs per QALY ranging from €5949 to €20 350 across countries [24]. Similarly, the cost per life-year with statins, a common therapy used in patients with Type 2 diabetes concomitantly with anti-hyperglycaemic agents to treat dyslipidaemia and reduce cardiovascular risk, has been estimated to range from £5400 to £13 300 for primary prevention and from £3800 to £9300 for secondary prevention. [25]

A limitation of this study is that the model used, like all models used to assess the long-term outcomes of patients with Type 2 diabetes, predicts long-term outcomes based on the results of short-term studies. However, the CORE Diabetes Model used here has been validated against published studies that had not been used to provide input data for setting up the model [13]. For each validation analysis, the progress of a patient cohort from a published epidemiological, clinical or modelling study was simulated, and the outcomes of the simulation were compared with those of the published study. The results indicated that the CORE Diabetes Model is capable of reliably predicting long-term patient outcomes.

In conclusion, this study investigated the cost–utility, in a UK setting, of liraglutide vs. glimepiride or sitagliptin (all added to metformin monotherapy), scenarios intended to simulate likely clinical practice in real life. The results suggest that liraglutide added to metformin monotherapy leads to improvements in quality adjusted-life expectancy and is a cost-effective option for the treatment of Type 2 diabetes in this setting.

## Competing interests

The study was funded by Novo Nordisk, the manufacturer of liraglutide. Novo Nordisk was responsible for running the model and analysing and reporting the results. The authors were responsible for the decision to submit the manuscript for publication. MJD has received advisory board and speaker and consultancy fees from Novo Nordisk and has received funding for research trials. BDC is employed by Novo Nordisk. ICS is employed by and owns shares in Novo Nordisk. WJV has received consultancy fees from Novo Nordisk.

## Abbreviations

NICE: National Institute for Health and Clinical Excellence
QALY: quality-adjusted life year

## Supporting information

**Table S1.** Summary of the costs of medicines and complications used in the model adjusted to 2008 costs.

**Table S2.** Summary of utilities and disutilities for the base case used in the model.

**Table S3.** Results of the base-case analysis by BMI subgroup in the liraglutide vs. sitagliptin comparison: quality-adjusted life years, costs and incremental cost-effectiveness ratios.

**Appendix S1.** Reference list.
