# Supplementary material for: Cost–utility analysis of liraglutide compared with sulphonylurea or sitagliptin, all as add-on to metformin monotherapy in Type 2 diabetes mellitus
Source: Diabet Med. 2012 Mar;29(3):313–20. doi: 10.1111/j.1464-5491.2011.03429.x (PMC3378710; doi:10.1111/j.1464-5491.2011.03429.x)
Supplement: Supplementary file 1 [file dme0029-0313-SD1.doc]

**References (Appendix)**

1. Clarke P, Gray A, Legood R, Briggs A, Holman R. The impact of diabetes-related complications on healthcare costs: results from the United Kingdom Prospective Diabetes Study (UKPDS Study No. 65). *Diabet Med* 2003; **20**: 442–450.

2. Currie CJ, Morgan CL, Peters JR. The epidemiology and cost of inpatient care for peripheral vascular disease, infection, neuropathy, and ulceration in diabetes. *Diabetes Care* 1998; **21**: 42–48.

3. UKPDS study group 40. Cost effectiveness analysis of improved blood pressure control in hypertensive patients with type 2 diabetes: UKPDS 40. UK Prospective Diabetes Study Group. *BMJ* 1998; **317**: 720–726.

4. Leese GP, Wang J, Broomhall J, Kelly P, Marsden A, Morrison W *et al*. Frequency of severe hypoglycemia requiring emergency treatment in type 1 and type 2 diabetes: a population-based study of health service resource use. *Diabetes Care* 2003; **26**: 176–1180.

5. Ghatnekar O, Persson U, Willis M, Ödegaard K. The cost-effectiveness of becaplermin (Regranex®) in the treatment of diabetic foot ulcers in four European countries. *Pharmacoeconomics* 2001; **19**: 767–778.

6. NHS Information Centre. Prescription Cost Analysis – England 2008. 2009. Available from: http://www.ic.nhs.uk/statistics-and-data-collections/primary-care/prescriptions/prescription-cost-analysis-2008 [accessed 14 January 2011].

7. Owens D, Barnett AH, Pickup J,Kerr D, Bushby P, Hicks D *et al.* Blood glucose monitoring in type 1 and type 2 diabetes: reaching a multidisciplinary consensus. Diabetes and Primary Care 2004; **6**: 8–16.

8. Lee AJ, Morgan CL, Morrissey M, Wittrup-Jensen KU, Kennedy-Martin T, Currie CJ. Evaluation of the association between the EQ-5D index (health related utility) and body mass index (Obesity) in hospital-treated people with type 1 diabetes, type 2 diabetes and with no diagnosed diabetes. *Diabetic Medicine* 2005; **22**: 1482–1486.

9. Tengs TO, Wallace A. One thousand health-related quality-of-life estimates. *Med Care* 2000; **38**: 583–637.

10. Palmer AJ, Roze S, Valentine WJ, Minshall ME, Foos V, Lurati FM *et al*. The CORE Diabetes Model: Projecting long term clinical outcomes, costs and cost-effectiveness of intervention in diabetes mellitus (types 1 and 2) to support clinical and reimbursement decision making. *Curr Med Res Opin* 2004; **20**(Suppl. 1): S5–S26.
