# Supplementary material for: Cost–utility analysis of liraglutide compared with sulphonylurea or sitagliptin, all as add-on to metformin monotherapy in Type 2 diabetes mellitus
Source: Diabet Med. 2012 Mar;29(3):313–20. doi: 10.1111/j.1464-5491.2011.03429.x (PMC3378710; doi:10.1111/j.1464-5491.2011.03429.x)
Supplement: Supplementary file 2 [file dme0029-0313-SD2.doc]

**Table A1.** Summary of the costs of medicines and complications used in the model adjusted to 2008 costs

| **Comparator price inputs (NHS price), including daily dose** | **Pack price** | **No. of days per pack** | **Price per day** | **Price per year** |
| --- | --- | --- | --- | --- |
| Metformin 2000 mg | £1.39 | 21 | £0.07 | £24.16 |
| SU (glimepiride) 4 mg | £5.11 | 30 | £0.17 | £62.17 |
| Sitagliptin 100 mg | £33.26 | 28 | £1.19 | £433.57 |
| Liraglutide 1.8 mg | £117.72 | 30 | £3.92 | £1432.26 |
| Liraglutide 1.2 mg | £78.48 | 30 | £2.62 | £954.84 |
| Insulin glargine (40IU) solostar* | £40.36 | 37.5 | £1.08 | £392.84 |
| **Self-monitoring of blood glucose^†#^** |  | ***No. per pack*** | ***Price per test*** |  |
| OneTouch Ultra (reagent strips) | £14.53 | 50 | £0.291 |  |
| Softclix lancets (0.4 mm/28 gauge) | £7.20 | 200 | £0.036 |  |
| **Direct costs (annual) of CVD complications** | **Cost** | **Reference** | | |
| MI 1st year | £4,914 | [1] | | |
| MI 2nd + years | £809 | [1] | | |
| Angina 1st year | £2,548 | [1] | | |
| Angina 2nd + years | £842 | [1] | | |
| CHF 1st year | £2,842 | [1] | | |
| CHF 2nd + years | £996 | [1] | | |
| Stroke 1st year | £3,006 | [1] | | |
| Stroke 2nd + years | £568 | [1] | | |
| Stroke death within 30 days | £3,791 | [1] | | |
| PVD 1st year | £2,618 | [2] | | |
| PVD 2nd + years | £2,618 | [2] | | |
| **Direct costs of renal complications** | **Cost** | **Reference** | | |
| Haemodialysis costs 1st year | £27,863 | [3] | | |
| Annual costs HD 2+ years | £27,863 | [3] | | |
| Peritoneal dialysis costs 1st year | £20,920 | [3] | | |
| Annual costs PD 2+ years | £20,920 | [3] | | |
| Renal transplant costs 1st year | £22,191 | [3] | | |
| Annual costs renal transplant 2+ years | £7,212 | [3] | | |
| **Direct costs of acute events** | **Cost** | **Reference** | | |
| Major hypoglycaemic event | £412 | [4] | | |
| **Direct costs of eye disease (event-based, except where stated)** | **Cost** | **Reference** | | |
| Laser treatment | £755 | [3] | | |
| Cataract operation | £1,740 | [1] | | |
| Follow-up from cataract operation | £118 | [1] | | |
| Blindness – year of onset | £977 | [1] | | |
| Blindness – following years (annual) | £315 | [1] | | |
| **Direct costs of neuropathy/foot ulcer** | **Cost** | **Reference** | | |
| Neuropathy 1st year | £1,066 | [2] | | |
| Neuropathy 2nd + years (annual) | £1,066 | [2] | | |
| Amputation (event-based) | £9,832 | [2] | | |
| Amputation prosthesis (event-based) | £625 | [2] | | |
| Gangrene treatment (annual) | £2,308 | [5] | | |
| Infected ulcer | £1,437 | [5] | | |
| Standard uninfected ulcer | £1,402 | [5] | | |

*Patients were switched to insulin glargine after the 5-year treatment period to replicate clinical practice.
**^†^**For blood glucose monitoring, costs of the most frequently prescribed products are used [6].
CHF, congestive heart failure; GRP, gross renal proteinuria; HD, haemodialysis; MA, microalbuminuria; MI, myocardial infarction; NHS, National Health System; PD, peritoneal dialysis; PVD, peripheral vascular disease.
^#^ The frequency of blood glucose testing used in the modelling was based on a consensus paper from Owens et al. [7], with three tests per week (£51.09 per annum) for patients using a regimen that incorporated a sulphonylurea, seven tests per week (£119.21 per annum) for patients using a regimen that incorporated insulin plus OADs and no tests per week (£0.00 per annum) for all other regimens.
