# Supplementary material for: Cost–utility analysis of liraglutide compared with sulphonylurea or sitagliptin, all as add-on to metformin monotherapy in Type 2 diabetes mellitus
Source: Diabet Med. 2012 Mar;29(3):313–20. doi: 10.1111/j.1464-5491.2011.03429.x (PMC3378710; doi:10.1111/j.1464-5491.2011.03429.x)
Supplement: Supplementary file 3 [file dme0029-0313-SD3.doc]

**Table A2.** Summary of utilities and disutilities for the base case used in the model

| **Event/state** | **Utility** | **Disutility** | **Reference** |
| --- | --- | --- | --- |
| Diabetes, no complications | 0.814 | – | [1] |
| Change in 1 unit BMI (kg/m^2^) | – | −0.01 | [7] |
| Angina | 0.682 | – | [1] |
| Congestive heart failure | 0.633 | – | [1] |
| Myocardial infarction, year of event | – | −0.129 | [1] |
| Myocardial infarction, 2+ years after event | 0.736 | – | [1] |
| Stroke, year of event | – | −0.181 | [1] |
| Stroke, 2+ years after event | 0.545 | – | [1] |
| Peripheral vascular disease | 0.570 | – | [8] |
| Microalbuminuria | 0.814* | – | [1] |
| Gross proteinuria | 0.814* | – | [1] |
| Haemodialysis | 0.490 | – | [8] |
| Peritoneal dialysis | 0.560 | – | [8] |
| Kidney transplant | 0.762 | – | [8] |
| Background diabetic retinopathy | 0.814* | – | [1] |
| Cataract | 0.794 | – | [9] |
| Macular oedema | 0.794 | – | [9] |
| Proliferative diabetic retinopathy | 0.794 | – | [9] |
| Severe vision loss/blindness | 0.734 | – | [1] |
| Neuropathy | 0.624 | – | [9] |
| Active ulcer | 0.600 | – | [9] |
| Healed diabetic ulcer | 0.814* | – | [1] |
| Amputation, year of event | – | −0.109 | [1] |
| Amputation, 2+ years after event | 0.680 | – | [1] |
| Major hypoglycaemic events | – | −0.0118 | [2] |
| Minor hypoglycaemic events | – | −0.0035 | [2] |

*No state-specific health utility identified – conservatively assumed to be equivalent to complication-free utility.
BMI, body mass index.
