# Supplementary material for: Cost–utility analysis of liraglutide compared with sulphonylurea or sitagliptin, all as add-on to metformin monotherapy in Type 2 diabetes mellitus
Source: Diabet Med. 2012 Mar;29(3):313–20. doi: 10.1111/j.1464-5491.2011.03429.x (PMC3378710; doi:10.1111/j.1464-5491.2011.03429.x)
Supplement: Supplementary file 4 [file dme0029-0313-SD4.doc]

**Table A3.** Results of the base-case analysis by BMI subgroup in the liraglutide vs. sitagliptin comparison: quality-adjusted life years, costs and incremental cost-effectiveness ratios

| **BMI ≥ 30 kg/m^2^** | **Liraglutide 1.2 mg + metformin** | **Liraglutide 1.8 mg + metformin** | **Sitagliptin (100 mg)  + metformin** |
| --- | --- | --- | --- |
| Life expectancy (years) | 11.946 (0.163) | 12.060 (0.160) | 11.765 (0.165) |
| Quality-adjusted life expectancy (years) | 7.295 (0.103) | 7.429 (0.100) | 7.059 (0.098) |
| Costs | £24,996 (556) | £26,428 (544) | £23,203 (564) |
| Δ life expectancy (years) | 0.181 (0.225) | 0.295 (0.217) | comparator |
| Δ quality-adjusted life expectancy (years) | 0.236 (0.140) | 0.37 (0.132) | comparator |
| Δ costs | £1,793 (779) | £3,225 (755) | comparator |
| Cost per life years gained | £9,884 | £10,932 | comparator |
| Cost per quality-adjusted life year | £7,593 | £8,721 | comparator |
| **BMI ≥ 35 kg/m^2^** | **Liraglutide 1.2 mg + metformin** | **Liraglutide 1.8 mg + metformin** | **Sitagliptin (100 mg)  + metformin** |
| Life expectancy (years) | 12.136 (0.158) | 12.314 (0.161) | 11.901 (0.152) |
| Quality-adjusted life expectancy (years) | 7.013 (0.096) | 7.199 (0.095) | 6.710 (0.090) |
| Costs | £25,846 (559) | £26,966 (554) | £23,990 (562) |
| Δ life expectancy (years) | 0.235 (0.212) | 0.413 (0.215) | comparator |
| Δ quality-adjusted life expectancy (years) | 0.303 (0.127) | 0.489 (0.126) | comparator |
| Δ costs | £1,856 (757) | £2,977 (790) | comparator |
| Cost per life years gained | £7,893 | £7,203 | comparator |
| Cost per quality-adjusted life year | £6,125 | £6,091 | comparator |

Data are mean (SD).

BMI, body mass index.
